# The Relationship between Unilateral Palatal Maxillary Canine Impaction and the Morphology of the Maxilla: A CBCT Study in Eastern Province of Saudi Arabia

**DOI:** 10.1055/s-0042-1757567

**Published:** 2022-12-28

**Authors:** Suliman Y. Shahin, Afsheen Tabassum, Arishiya Thapasum Fairozekhan, Ahmed Al Tuwaylib, Saeed Al-Sheyoukh, Shuaib Alzaher, Intisar Ahmad Siddiqui, Muhanad Alhareky

**Affiliations:** 1 Department of Preventive Dental Sciences, College of Dentistry, Imam Abdulrahman Bin Faisal University, Dammam, Kingdom of Saudi Arabia; 2 Department of Biomedical Dental Sciences, College of Dentistry, Imam Abdulrahman Bin Faisal University, Dammam, Kingdom of Saudi Arabia; 3 Department of Dental Education, College of Dentistry, Imam Abdulrahman Bin Faisal University, Dammam, Kingdom of Saudi Arabia

**Keywords:** CBCT, palatally impacted maxillary canine, morphology of the maxilla, canine impaction, retrospective study

## Abstract

**Objective** This study aimed to examine the correlation between the morphology of the maxilla and unilateral palatally impacted maxillary canine (PIMC) among the Saudi population in the Eastern Province of Saudi Arabia.

**Materials and Methods** In this retrospective study, 36 patients' records [(17 PIMC, 8 male, 9 female, mean age 16.75 ± 2.12 years) (19 control; 9 male,10 female, mean age: 17.16 ± 2.12 years)] were included from a teaching dental hospital. On cone-beam computed tomographic images, measurements of maxillary arch length (MAL), palatal vault depth (PVD), intermolar width, sum of widths of 4 maxillary incisors, available arch space (AAS), palatal maxillary width (PMW) in the molar and premolar regions, nasal cavity width (NCW), maxillary arch shape (MAS) (arch length/intermolar width x 100), and palatal vault shape (PVS) (the PVD/intermolar width x100) were performed. Data were analyzed by SPSS-20.0. *p* -value less than or equal to 0.05 reflected statistical significance.

**Results** This study's findings depicted that AAS ( *p* = 0.012), PVD ( *p* = 0.028), and PMW in the molar and premolar regions at the level of the alveolar crest ( *p* = 0.002 and *p* = 0.034) and mid-root ( *p* = 0.004 and *p* = 0.022) were significantly higher in the control compared to the PIMC group. PVS showed a significant difference between the PIMC and control groups ( *p* = 0.037). However, regarding MAS, no significant difference was observed ( *p* = 0.707).

**Conclusion** MAS was narrower in PIMC compared to the control group. The control group had a deeper palatal vault and greater AAS compared to the PIMC group. However, no significant difference was observed between groups regarding tooth size or NCW.

## Introduction

Maxillary canines are considered vital teeth in maintaining oral function, stability, and aesthetics. 1 The mean age of maxillary canine eruption is between 11 and 12 years for girls and between 12 and 13 years for boys. 2 Teeth eruption is a physiological process; however, occasionally a fully developed tooth remains embedded within soft or hard tissue after its natural eruption stage has passed, and it is considered impacted. 3 The maxillary canines are the most frequently impacted teeth after the third molars. 4 5 6 The prevalence of canine impaction in the maxilla has been stated to range between 1.7 and 2.2%, where palatal impaction accounted for about 85% of those impactions and buccal impaction consisted of 15%. 6 7 In the Arab population, a study has documented that impacted canine prevalence was 3.7%, and palatal impaction accounted for 69% of maxillary canine impactions. 8 In yet another study conducted in the Eastern region of Saudi Arabia, maxillary canines constituted 50.4% of total impacted teeth. 9

Several etiological elements have been suggested for maxillary canine impaction, including genetic susceptibility, 2 variation in maxillary arch length, 10 long path of maxillary canine eruption, 11 morphology of the lateral incisor, inadequate resorption of the primary root, and other dental anomalies. 2 Several treatment approaches have been recommended for the management of impacted maxillary canines, but timely diagnosis and the interception of potential impaction are the most appropriate strategy. 2

It has been reported that the etiological hypotheses of buccal and palatal maxillary canine impaction are significantly different. 12 Two major concepts have been suggested to clarify the incidence of palatal maxillary canine displacement. These theories are known as the “guidance theory” 13 and the “genetic theory.” 14 15 According to the guidance theory, the maxillary canine lacks the guidance for eruption due to local environmental factors such as odontomas, congenitally missing teeth, lateral incisor morphology, or supernumerary teeth. 2 13 The genetic theory delegates the occurrence of maxillary canine impaction to the developmental disruption of the dental lamina. 14 15 In another study, the palatally displaced canine was considered as an anomaly of genetic origin, as 33% of subjects with a palatally displaced canine were born with congenitally missing teeth. 16 Furthermore, the transverse maxillary arch width, particularly in the premaxillary region, is reduced in individuals with palatal maxillary canine impaction, especially in the premaxillary region. 17 However, Langberg and Peck observe any relationship between transverse maxillary width and canine impaction. 18 According to Schindel and Duffy, the discrepancy in maxillary transverse measurements increases the probability of maxillary canine impaction. 19

Several studies have been carried out to determine a correlation between the impacted maxillary canine position and the maxillary arch morphological characteristics, but there is a contradiction in the existing literature. 10 In a recent review of the literature, Ravi et al suggested that multiple linear and angular parameters calculated on various Two-dimensional (2D) radiographs such as lateral cephalograms, orthopantomograms, and posteroanterior cephalograms can be used to predict maxillary canine impaction. Three-dimensional studies, on the other hand, are required to precisely assess and diagnose canine impactions. 20 In addition, there is a substantial need to conduct further studies based on the ethnicity, race, and origin of the study population. 21 Therefore, further research is mandatory to find out the relationship between maxillary canine impaction and the morphology of the maxilla. In this study, our focus is on unilateral palatal canine impaction due to its high prevalence (85% of the total maxillary canine impaction). 7 Furthermore, it has been shown in the literature that orthodontic treatment of palatally impacted canines takes longer than controls with similar characteristics, and age of treatment is a risk predictor for the length of treatment. 22 The management and prognosis of impacted maxillary canines can vary based on the initial diagnosis and the location of the impacted canine, which in turn dictates management, ranging from a surgical vs a non-surgical approach to a single-arch versus double-arch treatment. 4 23 It has been found that impacted canines that underwent orthodontic treatment can display deleterious periodontal and pulpal indices when compared to controls. 23 In addition, it has been demonstrated that the location of impacted maxillary canines can have an effect on the severity of root resorption of neighboring teeth, which plays a pivotal role in the orthodontic and surgical management of the impaction. 24 Studies have shown that there is a high incidence of maxillary canine impaction in all regions of Saudi Arabia. 7 9 To date, no study has investigated a link between unilateral palatal maxillary canine impaction and maxillary arch morphology in the Saudi population. Therefore, this retrospective study was designed to examine the correlation between the morphology of the maxilla and unilateral palatal maxillary canine impaction among the Saudi population in the Eastern Province of Saudi Arabia aged between 13 and 22 years.

## Materials and Methods

### Ethical Approval

The ethical approval for this study was obtained from the Institutional Review Board of Imam Abdulrahman bin Faisal University (IAU) (IRB number: 2022-02-218). The study was conducted according to the guidelines of the Helsinki Declaration.

### Setting and Patients' Records

This study was a retrospective, teaching hospital-based study performed at Imam Abdulrahman bin Faisal University Dental Hospital. This study was carried out using the medical records of individuals (age group: 13–22) who visited the university's teaching dental hospital for orthodontic treatment between 2015 and 2021 in Dammam, Saudi Arabia. Patient inclusion criteria for the experimental group were (1) patient age: 13–22 years old, (2) no history of previous interceptive or orthodontic treatment, and (3) diagnosis by an orthodontist with unilateral palatal maxillary canine impaction. Inclusion criteria for the control group were patients requiring orthodontic and dentofacial orthopaedic treatment where the maxillary canines had fully erupted, whereas other inclusion criteria were similar to the study group. Patient exclusion criteria were (1) patient age: less than 13 or more than 22 years, (2) history of previous interceptive or orthodontic treatment, (3) presence of odontoma or supernumerary teeth, (4) presence of any congenital dentofacial anomaly (cleft lip or palate) or hereditary syndromes, (5) presence of multiple impacted teeth, (6) congenitally missing teeth, (7) skeletal dysplasia, and (8) bilateral palatal or buccal canine impactions.

### Sample Size Calculation

Sample size calculation was performed with an effect size of 0.7, at a significance level of 0.05 and a power of 80%. Taking into consideration the above parameters, the sample size was desired to be 16 subjects in each study group.

### CBCT Analysis

**Fig. 1:**
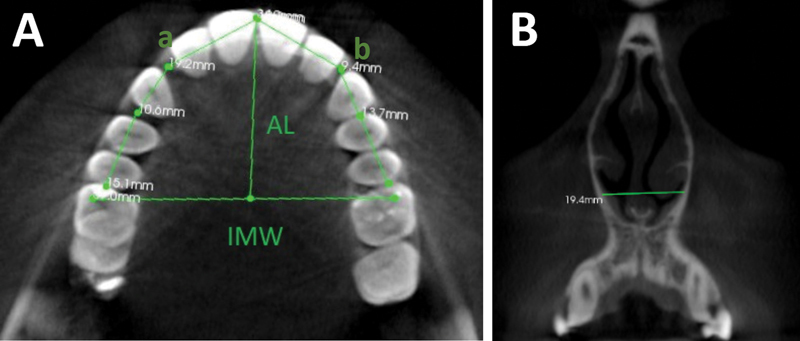
( **A** ) Representation of the measurements of the intermolar width (IMW); arch length (AL); available arch space; and sum of the maxillary four incisor (a distance of point a to b). ( **B** ) Representation of the measurements of the width of the nasal cavity.

**Fig. 2:**
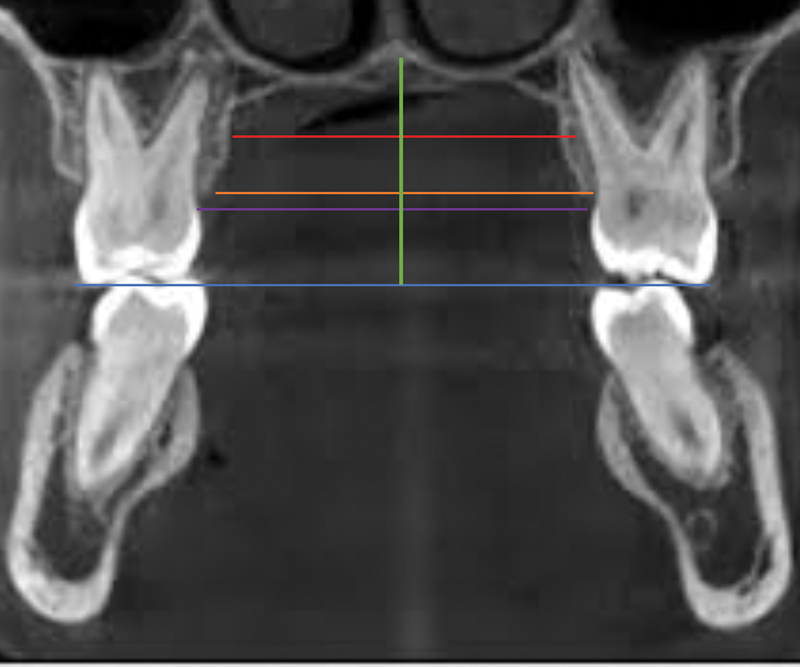
Representation of the measurements of the palatal vault depth (green line), with reference to occlusal plane (blue line); palatal maxillary width in molar region at the level of cement–enamel junction (purple line); at the alveolar crest level (orange line); and the mid-root level (red line).

Cone-beam computed tomography (CBCT) was performed as part of the standard orthodontic diagnostic records for patients undergoing orthodontic and dentofacial orthopaedic treatment at IAU Dental Hospital. Computerized tomographic imaging was carried out using a CS 9300 Premium imaging system (90 kV, 5 mA, 17 × 11 cm field of view, Carestream Dental, France). While taking the radiographs, patients were standing upright wearing a full lead apron with a neck collar, and they were asked to bite in a maximum intercuspation position with their chin centered within the chin rest base and their head supported with a 3D headrest. The image slice thickness taken was 0.75 mm. The following measurements were performed on the CBCT images by three different examiners: (1) maxillary arch length, (2) palatal vault depth, (3) intermolar width, (4) sum of 4 maxillary incisors' widths, (5) available arch space, (6) palatal maxillary width (PMW) in molar and premolar region (at cement–enamel junction [CEJ], alveolar crest and mid-palatal root level), and (7) width of nasal cavity. Interexaminer reliability and intraexaminer consistency were assessed at a 1-month interval based on 10% of the total sample data selected at random. All these measurements are depicted in Figs. 1 and 2 . A line was drawn between the mesiobuccal cusps of the right and left maxillary first molars. The length of this line was considered as intermolar width. A perpendicular line was drawn from the incisal edge of the upper central incisors to the straight line that determines intermolar width. This distance was recorded as the arch length. If any difficulty was encountered in the exact identification of the incisal edge position of the central incisors due to rotation or crowding, the measurement was recorded from the most labial side of the maxillary central incisor. The palatal vault depth was recorded as the vertical distance from the deepest point on the palatal vault to the horizontal contact line between the right and left first maxillary molars ( Fig. 1 ). The width of the nasal cavity was recorded at the broadest part of the lower third of the nasal cavity in the coronal section of the CBCT image. ICAT vision software (Science International, Q version 1.8.1.10, Imaging, Hatfield, Pennsylvania, United States) was utilized to record all the measurements. Each measurement was recorded twice, and the mean was used for statistical analysis.

The maxillary arch shape (arch length/intermolar width x 100) was determined by calculating the ratio of arch length to intermolar width measurements. The shape of the palate was evaluated by the ratio of the depth of the palatal vault to the intermolar width (palatal vault depth/intermolar width x100). The maxillary arch shape and the shape of the palate were compared between the control and palatally impacted canine groups.

### Statistical Analysis

Data analyses were performed using SPSS-20.0 (IBM, Chicago, Illinois, United States). Categorical data, including gender and systemic diseases, were presented as frequencies and percentages. A chi-squared test was used to compare these categorical variables between the control and palatally impacted maxillary canine (PIMC) groups. Numerical data based on measurements of CBCT are presented as mean ± standard deviation. These numeric variables were explored for the test of normality using the Kolmogorov–Smirnov test within the control and PIMC groups, which revealed a normal distribution. An unpaired t-test was used to compare mean differences between the control and PIMC groups. *p* -value less than or equal to 0.05 reflected statistical significance.

## Results

The 36 subjects included in this study were divided into two groups, that is, the PIMC group and the control group. The PIMC group consisted of 17 patients (8 male and 9 female) with a mean age of 16.75 ± 2.12 years. The control group consisted of 19 subjects (9 male and 10 female) with a mean age of 17.16 ± 2.12 years ( Table 1 ). The control and PIMC groups were statistically uniform concerning mean age ( *p* = 0.651) and gender ( *p* = 0.873). The reliability coefficient for items' measurement reliability was tested using Cronbach's alpha, which equaled 0.996, revealing highly reliable intrarater validity. Interrater consistency was calculated based on intraclass correlation, which equaled 0.985, also revealing a high degree of concordance between the examiners.

**Table 1:** Comparison of demographic characteristics between control versus PIMC group

Gender	Total ( *n* = 36)	Control ( *n* = 19)	Palatal ( *n* = 17)	*p* -value
o Male	17 (47.2%)	9 (47.4%)	8 (47.1%)	0.985
o Female	19 (51.4%)	10 (52.6%)	9 (52.9%)	

Abbreviation: PIMC, palatally impacted maxillary canine.

The available arch space ( *p* = 0.012) and the palatal vault depth ( *p* = 0.028) were significantly greater in the control group as compared to the PIMC group. However, no statistically significant differences were observed concerning the arch length, intermolar width, and sum of width of maxillary incisors between the two groups. PMW in the molar and premolar region at the level of the alveolar crest ( *p* = 0.002 and *p* = 0.034) as well as at the mid-root level ( *p* = 0.004 and *p* = 0.022) were significantly higher in the control group as compared to the PMIC group. Meanwhile, PMW in the molar region at the level of CEJ was marginally significant ( *p* = 0.070) and PMW in the premolar region at the level of CEJ was insignificant between the two groups ( *p* = 0.228). No statistically significant differences were observed concerning the width of the nasal cavity between the two groups ( Table 2 ).

**Table 2:** Comparison of maxillary arch parameters between the control and the PIMC groups

Variable	Control ( *n* = 19)	PIMC ( *n* = 17)	*p* -value
Maxillary Arch length	36.09 ± 4.62	35.72 ± 4.28	0.806
Available arch space	76.41 ± 5.37	71.35 ± 5.64	0.012 a
Sum 4 maxillary incisors	30.68 ± 2.77	30.93 ± 3.26	0.809
Palatal vault depth	21.78 ± 2.73	19.73 ± 2.48	0.028 a
Intermolar width	53.30 ± 4.47	53.10 ± 2.66	0.876
PMW in the molar region at CEJ	35.52 ± 4.27	33.29 ± 3.67	0.070
PMW in molar region at alveolar crest level	34.18 ± 3.95	29.91 ± 2.32	0.002 a
PMW in the molar region at the mid-root level	30.17 ± 3.25	26.96 ± 2.80	0.004 a
PMW in premolar region at CEJ	29.01 ± 2.56	27.52 ± 4.52	0.228
PMW in premolar region at alveolar crest level	27.71 ± 2.56	25.31 ± 4.23	0.034 a
PMW in premolar region at the mid-root level	24.26 ± 2.61	20.41 ± 6.33	0.022 a
Width of the nasal cavity	20.51 ± 3.39	21.66 ± 1.58	0.209

Abbreviations: CEJ, cement–enamel junction; PIMC, palatally impacted maxillary canine; PMW, palatal maxillary width.

a Significant at *p* ≤ 0.05.

In the same way, no statistically significant difference was seen between the two groups ( *p* = 0.707) with respect to the maxillary arch shape. However, regarding the palatal vault shape, a statistically significant difference was observed between the PIMC group and the control group ( *p* = 0.037) ( Table 3 ).

**Table 3:** Comparison of maxillary arch shape and palatal vault shape between the control and the PIMC groups

Variable	Control ( *n* = 19)	PIMC ( *n* = 17)	*p* -value
Maxillary arch shape 1	68.02 ± 9.38	67.15 ± 6.47	0.707
Palatal vault shape 2	41.14 ± 6.16	37.36 ± 5.81	0.037 ^a^

Abbreviation: PIMC, palatally impacted maxillary canine.

**1:** Measurements for the comparison of maxillary arch shape: arch length/intermolar width x 100. **2:** Measurements for the comparison of palatal vault shape: palatal vault depth/intermolar width x 100. ^a^ Significant at *p* ≤0.05.

## Discussion

The study aimed to determine whether there exist any correlations between palatally impacted canines and the morphology of the maxilla in the study population using CBCT analysis. The study design employed CBCT rather than conventional methods in the assessment and localization of palatally impacted canines. Dalessandri et al 25 emphasized that 3D-assessed CBCT indices are more reliable and far superior when compared to the 2D-based conventional assessments. It has also been reported that intra- and interobserver variability is very minimal when assessed by CBCT. 26 The mean age of the participants in our study was 16.75 years in the PIMC group with age-matched controls. This was consistent with the mean age found in previous literature reviews. 27

The most significant finding in our study was that both the available arch space and the palatal vault depth were significantly decreased in the PIMC subjects than in controls, prompting a narrower and shorter palate in the evaluated PIMC subjects. The decrease in the available arch space can be attributed to the posterior segment because the arch space was assessed by dividing the maxillary arch into four segments. The *p* -value was insignificant for the anterior segments (sum of the 4 maxillary anterior teeth), but the decreased overall available arch space showed a significant correlation in palatally impacted canine subjects than in controls, prompting a significant decrease in the posterior segments. Previous studies have found a strong association between intermolar width, available arch space, the sum of the anterior segments, and arch length being smaller in subjects with displaced maxillary canines than in controls. 10 28 29 But our study only showed a drastic decrease in the available arch space, followed by a minimal decrease in arch length, with no significant decrease in the sum of the anterior segments and intermolar width, contrary to the above studies. This could be ascribed to the sample size employed in our study.

Studies have also demonstrated marked transverse maxillary deficiency in the anterior segment in patients with canine impactions, which was not in agreement with our study. 17 The anterior segment comprising four maxillary incisors was wide enough but still associated with PIMC. This could be explained by studies that revealed that in about 85% of palatally impacted canines, sufficient space was available for eruption. 30 Arch length sufficiency was also reported by Stellzig et al in 82% of subjects with palatally displaced canines. 31 Hence, these findings were in agreement with our study, and a more predictable etiology for PIMC is the failure of the canine to migrate from the palatal to the buccal aspect, as explained by McSherry and Richardson. 32 Another possibility could be the anomalous lateral incisor failing to guide the canine in its vicinity, as reported by Becker et al. 12

In the current study, the intermolar width did not show any statistically significant results between controls and the PIMC group. The majority of the studies in the literature did not report any significant observations in intermolar width between PIMC and controls, 17 18 33 34 35 except for a couple of studies reported by Kim et al 29 and Schindel and Duffy. 19 The discrepancies can be attributed to the diverse methodologies and varied ethnicities included in the study populations. Further, none of the previous studies evaluated the transverse PMW dimensions at different anatomic reference points in the molar and premolar regions. This study showed a significant reduction in the transverse dimensions at the alveolar crest and mid-root region in PIMC subjects. This was in line with a similar observation by Elmarhoumy, who concluded a reduced maxillary transverse dimension in impacted canine patients when evaluated at four different anatomic reference points. However, the latter's reference points differed from those of the current study. 36 The clinical significance of these transverse dimensions when coupled with the assessment of arch length, perimeter, and crowding will assist the orthodontist in the appropriate selection of rapid maxillary expansion devices along with the prediction of relapse and retention. 37

The depth of the palatal vault differed significantly between the two study groups in this study. Kim et al 29 have extensively studied the depth of the palatal vault in palatally and buccally impacted canines and justified that a deeper and narrower palatal vault contributes to the increased vertical length of the maxilla, which thereby influences the further separation of the maxillary lateral incisor and the canine tooth germs, thereby contributing to the lack of guidance for the canine to erupt significantly. But our findings showed a shallow palatal vault in the palatally impacted canines group, which is not in agreement with Kim et al's report. 29 Furthermore, our study also showed an increase in transverse diameter in the anterior segment. When this finding is coupled with the shallow palatal vault in PIMC subjects, the pathogenesis for impaction can be deduced to be of genetic origin on the palatal aspect. 16 But our findings could not support the guidance theory in the etiology of PIMC as reported by similar study. 38

The palatal vault shape also showed significant observations in the control and experimental groups. But previous studies of unilaterally and bilaterally palatally impacted canines denoted no significant palatal vault morphology, and the observed subjects showed no maxillary transverse constriction except for a significant reduction in the inter-canine width between the two groups. 39

Our findings of decreased transverse palatal width and shallow palatal height substantiate the findings of Tang et al, who found a decreased palatal vault depth and palatal intermolar area when measured on casts of growing children with PIMC. 40 Our study shed some light on the effects of the transverse dimension of the palatal morphology at various levels of the teeth, such as the CEJ, the alveolar crest, and the mid-root level. Our findings suggest a more important role of the morphology of palatal bone compared to the teeth, as the PMW in the molar and premolar region at the level of the alveolar crest as well as at the mid-root level was significantly higher in the control group when compared to the PMIC group ( *p* < 0.05). Meanwhile, the PMW in the molar and premolar region at the CEJ was both statistically insignificant between the two groups ( *p* = 0.07 and *p* = 0.228, respectively).

The PIMC subjects in our study exhibited wide anterior transverse diameter, constricted posterior transverse diameter, decreased available arch space and palatal vault depth, and a marginal decrease in arch length and intermolar width. Of all the above, the constricted posterior transverse segment has a direct clinical implication. The constricted posterior segment in PIMC found in this study could be interrupted early by interceptive orthodontics in younger populations by rapid maxillary expansion. 28 Furthermore, managing impacted canines in younger individuals is more successful and requires fewer orthodontic visits compared to adults. 2 The current findings help the clinician to intercept at an early phase with some preventive protocols, which further prevent the complication of canine impactions. But a combination of transpalatal arch therapy and extraction of the deciduous canine in the late mixed dentition period has proved to be more effective in reducing the risk of palatal impactions. 41 Perhaps utilizing the findings from CBCT imaging in the early phases of PIMC can aid clinicians in treatment decisions regarding altering the maxillary width.

## Clinical Implication

The findings of this study depicted that the maxillary arch's morphological characteristics might be used as a risk indicator for the early detection and diagnosis of maxillary canine impaction in the Saudi population. Early diagnosis using preoperative radiographic assessment via CBCT scans yields more benefits and reduces the patient's treatment burden through early intervention. 10 Rapid maxillary expansion at an early age might contribute to decreased incidence of PIMC. The data from the current study on maxillary arch width, available arch space, and palatal vault depth would be useful for early prediction of a PIMC during the initial diagnosis and decision-making phase. By comparing these parameters to normal features of maxillary morphology, early detection of disruption in maxillary canine eruption could be identified, and orthodontists' treatment planning could be improved. Furthermore, patients and their families could be well informed with respect to the most suitable treatment options. 2

## Limitations of the Study

The main limitations of this study were that the sample size used was not large enough and the study's focus was on only one ethnic population. The PIMC subjects in our study were restricted to unilateral palatal canine impactions, and the influence of gender on maxillary morphology was not deduced. Despite the limited sample size, this study improves the existing evidence regarding the relationship between palatally maxillary impacted canines and the morphology of the maxilla.

Therefore, future studies should be conducted at a multicenter level, with the goal of comparing these parameters across various racial and ethnic groups. Furthermore, evaluating the longitudinal effect of early intervention with rapid maxillary impaction on the prognosis of impacted maxillary canines could yield some valuable clinical implications.

## Conclusion

This study established a correlation between unilateral palatal maxillary canine impaction and the morphology of the maxilla in the Saudi population as follows:

1. The most significant findings in the current study were decreased available arch space and palatal vault depth, indicating a constricted posterior transverse segment and a shallow palate in the PIMC group.
2. The PIMC subjects exhibited wider anterior transverse diameter when compared to their posterior counterparts. The constricted posterior segment has a direct clinical implication, which, when intercepted early, can reduce the risk of palatal impactions.

**Abbreviations**
(PIMC): Unilateral palatally impacted maxillary canine
(MAL): Maxillary arch length
(PVD): Palatal vault depth
(IMW): Intermolar width
(SFMI): Sum of widths of 4 maxillary incisors
(AAS): Available arch space
(PMW): Palatal maxillary width
(NCW): Nasal cavity width
(MAS): Maxillary arch shape, and
(PVS): Palatal vault shape
